# Network Pharmacology–Based Identification of Key Mechanisms of Xihuang Pill in the Treatment of Triple-Negative Breast Cancer Stem Cells

**DOI:** 10.3389/fphar.2021.714628

**Published:** 2021-10-19

**Authors:** Yu-Zhu Zhang, Jia-Yao Yang, Rui-Xian Wu, Chen Fang, Hai Lu, Hua-Chao Li, Dong-Mei Li, Hua-Li Zuo, Li-Ping Ren, Xiao-Yuan Liu, Rui Xu, Jia-Huai Wen, Hsien-Da Huang, Ri Hong, Qian-Jun Chen

**Affiliations:** ^1^ National Resource Center for Chinese Materia Medica, China Academy of Chinese Medical Sciences, Beijing, China; ^2^ Breast Department, Guangdong Provincial Hospital of Chinese Medicine, Guangzhou, China; ^3^ School of Chinese Medicine, Hong Kong Baptist University, Hong Kong, China; ^4^ Breast Department, Maternal and Child, Health Hospital of Sanya, Sanya, China; ^5^ Zhuhai Hospital of Guangdong Provincial Hospital Medicine, Zhuhai, China; ^6^ Warshel Institute for Computational Biology, The Chinese University of Hong Kong, Shenzhen, China; ^7^ School of Life and Health Sciences, The Chinese University of Hong Kong, Shenzhen, China; ^8^ School of Computer Science and Technology, University of Science and Technology of China, Hefei, China

**Keywords:** TNBC, cancer stem cell, network pharmacology, NR3C2, Xihuang pill, naringenin

## Abstract

Xihuang pill, an approved Chinese medicine formula (state medical permit number. Z11020073), is a commonly used adjuvant drug for cancer patients in China. Xihuang pill has a satisfactory effect in treating breast cancer in clinics, especially triple-negative breast cancer (TNBC), which is the most aggressive type of breast cancer, and finite effective therapies. However, the mechanism of Xihuang pill in treating TNBC remains unclear. The present study aims to explore the pharmacological mechanism of Xihuang pill in treating advanced TNBC. We identified the main chemical components of Xihuang pill by using HPLC-Q-TOF-MS/MS. The 3-(4,5-dimethylthiazol-2-yl)-2,5-diphenyltetrazolium bromide (MTT) analysis shows that serum containing Xihuang pill (XS) had no obvious killing effect on any subtype of breast cancer cells, but it inhibited mammosphere colony formation of two TNBC cell lines (4T1 and HCC1806 cells) and could enhance the inhibitory effect of paclitaxel (PTX) on the proliferation of 4T1 and HCC1806 cells when combined with PTX. Seventy-six active compounds in Xihuang pill, their 300 protein targets, and 16667 TNBC stem cell–related genes were identified. The drug–herb–active compound–target gene–disease network and enrichment analyses were constructed with 190 overlapping candidate targets. Through text mining and molecular docking, the target gene NR3C2 and its active compound naringenin were selected for further validation. According to the TCGA database, we observed that a high expression of NR3C2 promoted a higher survival probability regarding overall survival (OS). *In vitro* experiments indicated that naringenin presented an identical effect to XS, possibly by regulating the NR3C2 expression. Overall, this study explored the effect of Xihuang pill in treating advanced TNBC cells and showed that naringenin, which is the key active compound of Xihuang pill, could lessen the stemness of TNBC cells to produce a synergistic effect on PTX by regulating the NR3C2 gene.

## Introduction

Traditional Chinese medicine (TCM), which is a significant part of alternative and complementary medicine, has been clinically used in Asia for more than a 1,000 years (Zhang Y. F. et al., 2019). As important TCM treatment methods, herbs and formulas have a positive clinical effect on cancer treatment, especially as adjuvant treatments, which cooperate with Western medicines to enhance the sensitivity of anticancer drugs and reduce side effects (Park et al., 2013; Meng et al., 2017; Huang et al., 2019). Xihuang pill is an approved Chinese medicine formula (state medical permit number Z11020073) that consists of calculus bovis (a Chinese mineral medicine named Niu Huang, which are the dried gallstones of *Bos taurus domesticus* Gmelin), Myrrha [the dried resin of *Commiphora myrrha* (T. Nees) Engl., Mo Yao in Mandarin], Olibanum (the dried resin of *Boswellia carterii* Birdw., Ru Xiang in Mandarin), and Moschus (the dried secretion of mature male sachet, namely, She Xiang, which is a traditional medicine derived from animals). Xihuang pill has been documented in the *Wai Ke Quan Sheng Ji* as a secret recipe of Wang Hongxu in the Qing Dynasty (Guo et al., 2015). It has been used to treat liver cancer (Feng et al., 2020), colorectal cancer (Yu and An, 2017), cervical cancer (Chang, 2017), and other malignant tumors in clinics in China. Qian et al. reported that Xihuang pill combined with TP chemotherapy had a significant clinical effect in treating advanced breast cancer, where the total effective rate of the Xihuang pill adjuvant group was higher than that of the TP chemotherapy group (Qian et al., 2020). Wang et al. reported that the Xihuang pill–combined group also has a satisfactory effect in the treatment of advanced breast cancer with improvements in the survival rate and life quality, which has a certain clinical application value (Wang and Yang, 2019). However, the current study of Xihuang pill remains in the basic stage, and the drug targets and pharmacological mechanisms of Xihuang pill in treating triple-negative breast cancer (TNBC) are still unclear.

Breast cancer is the most common malignancy found in women throughout the world (Bray et al., 2018). TNBC, which is the most aggressive subtype of breast cancer without an expression of the progesterone receptor, estrogen receptor, and human epidermal growth factor receptor 2, accounts for 10–20% of all breast cancers (Venkitaraman, 2010; Hudis and Gianni, 2011). Compared with other breast cancer subtypes, TNBC presents a highly invasive nature with a higher risk of distant metastasis, a higher rate of recurrence, and a shorter overall survival (OS) rate in the metastatic setting (Foulkes et al., 2010; Morante et al., 2018). Studies have shown that tumor metastasis and recrudescence, which are highly relevant to cancer stem cells (CSCs), are the major causes of death in TNBC (Nguyen et al., 2009; Doherty et al., 2017; Morante et al., 2018). The CSCs are a group of stem-like neoplastic cells with self-renewing and tumor-initiating properties (Reya et al., 2001). The effective treatment of TNBC is confined to surgery, radiotherapy, and chemotherapy and lacks target therapy (De Giorgi et al., 2007). The effect of standard chemotherapy for TNBC is confined by the existence of the CSCs (Mani et al., 2008). Thus, it is essential to improve the effectiveness of current drugs and search for new drugs that target TNBC stem cells.

**FIGURE 1:**
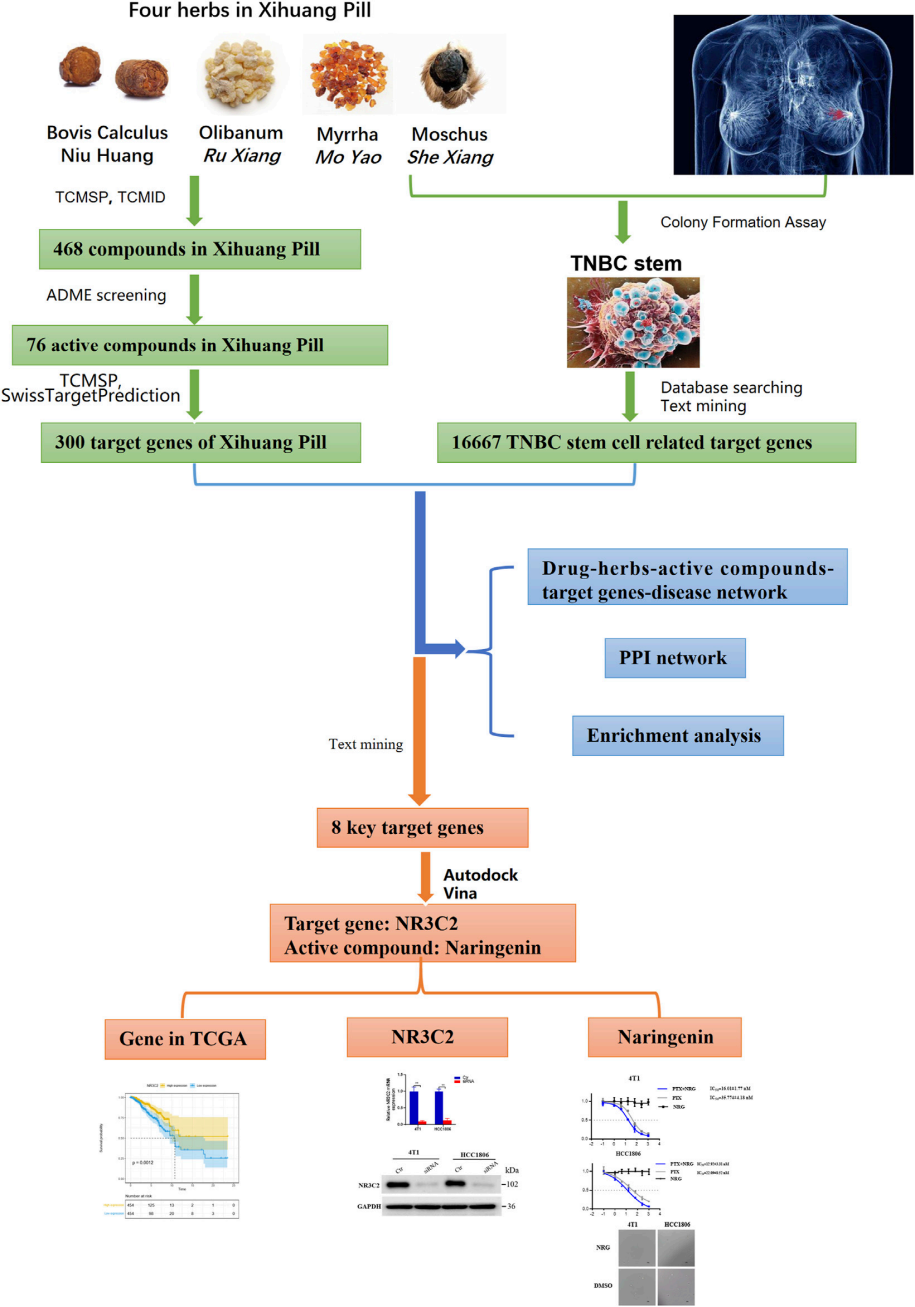
Workflow of this study.

Network pharmacology was first advanced by Andrew L. Hopkins. It is based on network theory and systems biology and is a practical tool to explore the mechanisms of drug action and discover novel drugs (Hopkins, 2008; Berger and Iyengar, 2009). Network pharmacology contests the conventional notion of “one disease, one target, one drug” and proposes the new concept of a “multicomponent, multi-target network,” which is consistent with the characteristics of complex compositions, multiple targets, and diversified regulatory approaches of Chinese medicine formulas (Chen et al., 2014; Chandran et al., 2015; Chandran et al., 2017). Therefore, network pharmacology provides a novel approach to explore the action of Chinese medicine formulas. Nonetheless, due to various qualities of databases and network algorithms and the lack of unified standards, the accuracy of the network pharmacology analysis results remains limited (Zhang R. et al., 2019). Hence, in this study, we used both network pharmacology–related bioinformatics tools to predict the active compounds, target proteins, and pathways in Xihuang pill on targeting TNBC stem cells and systems biology results in selecting active compounds and target proteins. Molecular docking, text mining, and *in vitro* experiments were also performed to validate the results. The flow chart is presented in Figure 1.

## Materials and Methods

### Cell Lines and Culture Conditions

Breast cancer cell lines T47D, MCF-7, SKBR3, 4T1, and HCC1806 were purchased from the American Type Culture Collection (ATCC; Manassas, VA), cultured in Dulbecco's Modified Eagle Medium (DMEM) supplemented with 10% fetal bovine serum, and incubated in a moistened atmosphere of 37°C and 5% CO_2_ (WCI-180, Wiggens, Germany). All cell culture reagents were obtained from Thermo Fisher Scientific (Gibco, United States).

### Qualitative HPLC-Q-TOF-MS/MS Analysis of Xihuang Pill

HPLC-Q-TOF-MS/MS was utilized to qualitatively determine the constituents of Xihuang pill*.* Two grams of the drug was placed into a 10-ml glass test tube, added to 8 ml of 50% methanol–water solution to soak for 4 h, and subsequently, ultrasonicated at 40°C for 40 min and at 1,300 r/min for 10 min, respectively. Before the HPLC analysis was performed, the supernatant solution was filtered through a Millipore filter with a pore size of 0.22 μm. Then, a total volume of 10 μl was injected.

Total ion chromatograms (negative mode) and the identified constituents of Xihuang pill are shown in Supplementary Figure S1.

### Drug and Animal Serum Preparation

Female Sprague–Dawley rats were acquired from the Experimental Animal Center of Southern Medical University (Guangzhou, China). Xihuang pill was obtained from Guangdong Provincial Hospital of Chinese Medicine (batch number: 6938706201176; specification: 0.1 g × 30 bottles/box; the composition ratio of Moschus, calculus bovis, Myrrha, and Olibanum was 15 : 15 : 550 : 550, respectively). The drug was dissolved in normal saline to prepare a 0.0625 g/ml suspension. The conversion coefficient between adult humans and rats being 6.25, the dosage to humans is 6 g/60 kg and that to rats is 0.625 g/kg. The rats were given intragastric administration of Xihuang pill at 10 ml/kg once a day for 14 consecutive days. After 14 days, 4 h after the last intragastric administration of Xihuang pill, the blood of the rats was collected from the abdominal aorta by intraperitoneal injection of chloral hydrate. After left standing at room temperature for 2 h, the serum was separated by centrifugation and immersed in a water bath at 56°C for 30 min. Finally, a 0.22-μm microporous filter was used for sterilization, and the serum was frozen at −20°C. All experiments in animals were conducted in conformity to the relevant institutional regulations and guidelines and were permitted by the Animal Experiment Committee of Guangdong Provincial Hospital of Chinese Medicine (No. 2020030).

### Cell Viability Assay

The cells at a density of 5,000 cells per well were seeded in 96-well plates and put into an incubator at 37°C and 5% CO_2_ overnight. Then, the cells were treated with reagents for 48 h. Cell viability was investigated using an MTT [3-(4,5-dimethylthiazol-2-yl)-2,5-diphenyl tetrazolium bromide] assay (Sigma-Aldrich, Saint Louis, MO, United States), and the absorbance was measured at 490 nm. Three repeated experiments were performed. The reagents included serum with or without Xihuang pill, paclitaxel (PTX) at a concentration gradient of 0, 0.1, 1, 4, 16, 64, 256, and 1024 nM with or without serum containing Xihuang pill (XS) and 0, 0.1, 1, 4, 16, 64, 256, and 1024 nM PTX with or without 0, 0.1, 1, 4, 16, 64, 256, and 1024 nM naringenin, or 0, 0.1, 1, 4, 16, 64, 256, and1024 nM of only naringenin. Xihuang pills (state medical permit number Z11020073) and PTX were purchased from Guangdong Provincial Hospital of Chinese Medicine, and naringenin, purity 98%, was obtained from Nanjing Spring and Autumn Biological Engineering Co., Ltd.

### 3D Induction of Stem Cell Assay

Fibrinogen was diluted to 2 mg/ml with T7 buffer. The cells were prepared for suspension at a concentration of 1 × 10^4^/ml and mixed with fibrinogen (2 mg/ml) at a ratio of 1:1 at 5,000 cells per ml and 1 mg per ml fibrinogen in the mixture of fibrin gels. In each well of a 96-well plate, 1 µl thrombin was added and mixed with a 50 μl cell/fibrinogen mixture. The 96-well plate was placed in the incubator at 37°C with 5% CO_2_ for 30 min. Then, 0.2 ml of the culture medium was added, and the cells were transferred back into the incubator.

### Mammosphere Colony Formation Assay

The cells at a concentration of 10,000 cells per ml were cultured in DMEM without serum in ultralow attachment plates and incubated at 37°C with 5% CO_2_ for 10 days. Then, the observations and photographs of the formed mammospheres were downloaded under a microscope.

### Identification and Prediction of Gene Targets for Active Compounds in Xihuang Pill

The chemical compounds Niu Huang ( calculus bovis), Mo Yao (Myrrha), Ru Xiang (Olibanum), and She Xiang (Moschus) were collected from the traditional Chinese medicine systems pharmacology database (TCMSP) (Version 2.3) (http://lsp.nwsuaf.edu.cn/) (Ru et al., 2014) and the traditional Chinese medicine integrative database (TCMID) (http://www.megabionet.org/tcmid/, Xue et al., 2013). Then, the TCMSP and SwissADME database (http://www.swissadme.ch, Daina et al., 2017) were used to select the chemical compounds with satisfactory pharmacokinetic properties through ADME screening. In the TCMSP, the chemical compounds were selected depending on oral bioavailability (OB) and drug-likeness (DL). Compounds with higher OB indicate that more effective components can arrive in the circulation. Compounds with OB ≥ 30% are considered to have high OB. A high DL implies that the compounds are more likely to be drugs. Compounds with DL ≥ 0.18 were considered to have high DL. In the SwissADME database, human gastrointestinal absorption (HIA) and DL were used to screen the chemical compounds. Chemical compounds with high HIA and more than two out of rule-of-five [Lipinski (Pfizer), Ghose (Amgen), Veber (GSK), Egan (Pharmacia), and Muegge (Bayer)] were collected as active compounds.

The protein targets of the active compounds in Xihuang pill were predicted by the TCMSP and SwissTargetPrediction database (http://www.swisstargetprediction.ch/, Daina et al., 2019). The targets of Niu Huang ( calculus bovis), Ru Xiang (Olibanum), and Mo Yao (Myrrha) were predicted by the TCMSP. For She Xiang (Moschus), the small molecular structures were collected from the PubChem database (https://pubchem.ncbi.nlm.nih.gov/, Kim et al., 2016). Then, the targets of the active compounds were predicted using the SwissTargetPrediction database. Finally, all target information was standardized using the UniProt database (http://www.UniProt.org/, Bateman et al., 2015).

### Collection of Gene Targets for TNBC Stem Cells

TNBC stem cell–related genes were collected from two databases: GeneCards (https://www.genecards.org/, Stelzer et al., 2016) and Online Mendelian Inheritance in Man (OMIM) (https://www.omim.org/, Kahl, 2015) by searching the keyword “TNBC stem cell.” After deleting duplicate genes, we collected the overlapping target genes related to TNBC stem cells as the candidate targets. In addition, text timing was performed to improve the accuracy of the collection of TNBC stem cell–related target genes. Jiang et al. classified TNBCs into four transcriptome-based subtypes, and the mesenchymal-like (MES) subtype was enriched in the mammary stem cell pathways (Jiang et al., 2019). Therefore, the gene sets of the MES subtype were collected, and the genes in the gene sets were gathered from the Gene Set Enrichment Analysis (https://www.gsea-msigdb.org/gsea/index.jsp, Subramanian et al., 2005). After deleting duplicate genes, we collected the overlapping target genes related to TNBC stem cells as the candidate targets.

### Network Construction and Analyses

In this study, network pharmacology was used to explore the interrelationships of Xihuang pill, herbs, active compounds, target genes, and TNBC stem cells. To characterize the mechanism of Xihuang pill on TNBC stem cells, a drug–herb–active compound–target gene–disease network was generated using Cytoscape 3.5.0 (Su et al., 2014).

### Enrichment Analyses

The Gene Ontology (GO) functional enrichment analysis and Kyoto Encyclopedia of Genes and Genomes (KEGG) pathway enrichment analysis were performed by the database for annotation, visualization, and integrated discovery (DAVID; Version 6.8) (https://david.ncifcrf.gov/, Huang et al., 2009). The GO terms were classified into three categories: molecular function (MF), biological process (BP), and cellular component (CC). Additionally, KEGG analysis results were input into OmicShare Tools (https://www.omicshare.com/) to draw the dot plot.

### Metaplastic Breast Cancer–Related Systems Biology Results

The gene expression microarray data (GSE10885) of patients with metaplastic breast cancer and patients with common breast tumors were derived from the National Center for Biotechnology Information (NCBI) and Gene Expression Omnibus (GEO) (https://www.ncbi.nlm.nih.gov/), in which the significant genes of metaplastic breast cancer had stem cell–like characteristics (Hennessy et al., 2009).

### Molecular Docking Simulation

First, the 3D structures of the ligand molecules were obtained from the ZINC database (http://zinc.docking.org/, Sterling & Irwin, 2015), and the Mol2 files were saved. The crystal structures of the target proteins, which were collected from the RCSB Protein Data Bank (PDB) database (http://www.rcsb.org/, Rose et al., 2017), were saved as PDB files. Then, the ligand molecules for docking were prepared by PyMol, and the target proteins and ligand molecules were edited and saved through Autodock Tools in PDBQT formats (Seeliger and De Groot, 2010; Morris et al., 2010). Autodock vina 1.1.2 (http://vina.scripps.edu/, Trott & Olson, 2019) was used to estimate the binding ability of the molecules and targets. After docking, the results were selected for further study according to the affinity score and text mining.

### NR3C2 Knockdown

4T1 and HCC1806 cell lines were transfected with siRNA using Lipofectamine 2000 (Invitrogen) following the protocol from the manufacturer. Silencer siRNA (RIBOBIO, siG000004306B-1–5) or negative control siRNA was used in RNA interference experiments. The sequences of NR3C2 siRNAs are GUG​GAU​AUA​UUU​ACC​UGG​ATT. After the transfected cells were incubated for 48 h, the efficiency of the NR3C2 knockdown was verified by RT-qPCR and WB.

### RNA Extraction and Quantitative Real-Time PCR

Total RNA was extracted using the TRIzol reagent (Takara, Beijing, China) and reverse transcribed into cDNA. Then, TB Green^TM^ Premix Ex TaqTM II (Takara, Beijing, China) with a CFX96^TM^ Real-Time System (ABI Quant Studio seven Flex, Applied Biosystems, United States) was used to measure gene expression levels. The expression level of glyceraldehyde-3-phosphate dehydrogenase was used as the control, and the 2^(−△△Ct)^ method was conducted to analyze the relative mRNA levels.

### Western Blot Analyses

To quantify the expression levels of NR3C2, the cells were lysed with RIPA buffer, separated by SDS–PAGE gels, transferred onto polyvinylidene difluoride membranes (ISEQ00010, Millipore, United States), and visualized by an enhanced chemiluminescent reagent on a multifunctional imaging system (Tanon 5200).

### Statistics

Data are presented as mean ± SD (standard deviation). Statistical analyses were conducted using GraphPad Prism (Version 8, San Diego, CA, United States), and Student's *t*-test was used to examine the significance of a difference among the groups. *p* values below 0.05 were considered statistically significant.

## Results

### Effect of XS on Breast Cancer Cell Lines

**FIGURE 2:**
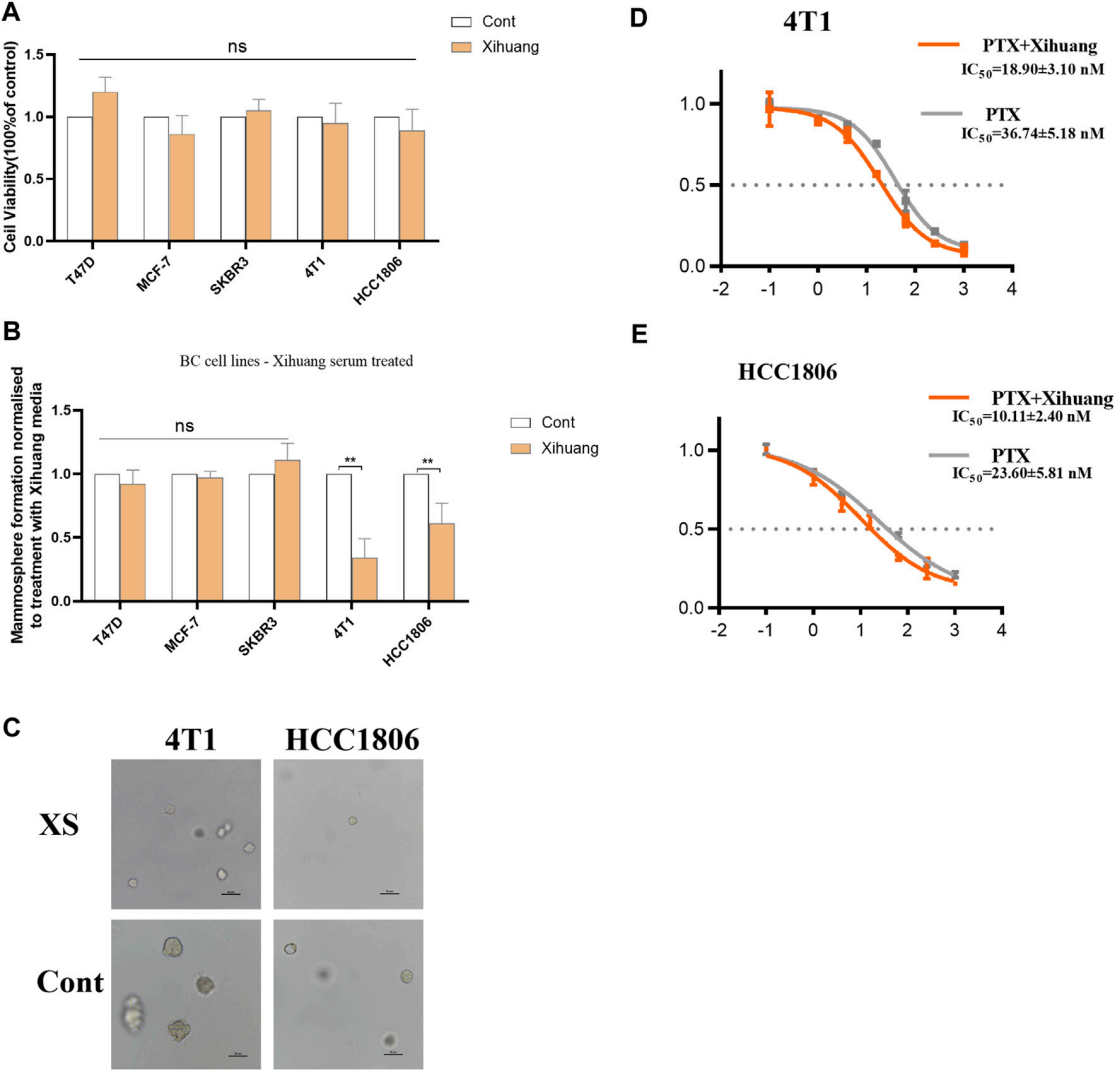
Effect of serum containing Xihuang pills (XS) on stem cell colony formation of TNBC cells. **(A)** MTT analysis indicated that the proliferation of breast cell was not inhibited by XS treatment in 48 h. **(B)** XS increased mammosphere colony formation in TNBC cell lines (4T1 and HCC1806) compared to control serum (CONT). **(C)** Representative photomicrographs of mammospheres formed by 4T1 and HCC1806 cells treated with serum containing Xihuang pills and control serum (CONT) are shown. Scale bar 50 µM. **(D,E)** 4T1 and HCC1806 cells were treated with 0, 0.1, 1, 4, 16, 64,256, or 1024 nM PTX for 48 h. MTT assay was used to detect the viability of cells (XS compared to CONT). *p < 0.05, **p < 0.01, ***p < 0.001 vs the control group.

The effect of XS on breast cancer was detected by using MTT analysis. As the results have shown, compared with the control groups, the viabilities of T47D, MCF-7, SKBR3, 4T1, and HCC1806 cells treated with XS for 48 h were not significantly different (*p* > 0.05), which indicates that treatment with Xihuang pill cannot directly inhibit the proliferation of breast cells (Figure 2A). For further study, a colony formation assay was conducted. In the common breast cancer groups (T47D, MCF-7, and SKBR3 cells), there was no significant difference in mammosphere colony formation between the control group and the group with XS (*p* > 0.05). In contrast, in the TNBC cell groups (4T1 and HCC1806 cells), XS decreased the formation of mammosphere colonies (*p* < 0.01). This outcome shows that XS suppressed mammosphere colony formation in TNBC cell lines (Figure 2B). The representative photomicrographs of mammospheres formed by 4T1 and HCC1806 cells treated with XS and control serum further and more vividly display the inhibition of mammosphere colony formation on two TNBC cell lines by XS (Figure 2C). The MTT assay was used to detect the effect of PTX with XS. For 4T1 cells, the IC_50_ value of PTX in the XS group was 18.90 ± 3.10 nM, while the IC_50_ value of the PTX-only group was 36.74 ± 5.18 nM (Figure 2D). For HCC1806 cells, the IC_50_ value of PTX with XS was 10.11 ± 2.40 nM, while the IC_50_ value of PTX-only group was 23.60 ± 5.81 nM (Figure 2E). The outcomes indicated that the PTX with XS groups had lower IC_50_ values than the PTX-only groups for both 4T1 and HCC1806 cells. Thus, the combination of XS and PTX can enhance the effect of PTX in inhibiting the proliferation of 4T1 and HCC1806 cells (Figures 2D,E). Although XS cannot directly kill breast cancer cells, it can constrain the mammosphere colony formation of TNBC cells and enhance the efficacy of PTX on TNBC cells.

### Active Compounds in Xihuang Pill and Candidate Targets

First, 468 chemical compounds in Xihuang pill were collected from the TCMSP and TCMID. Through ADME screening, 76 compounds with OB ≥ 30% and DL index ≥0.18 or high GI absorption and more than two drug-likeness (Lipinski, Ghose, Veber, Egan, and Muegge) were selected for further study (Table 1).

**TABLE 1:** The active compounds in Xihuang Pill.

No	Mol Id	Compounds	Herbs	OB	DL
1	MOL008838	methyl (4r)-4-[(3r,5s,7s,8r,9s,10s,12s,13r,14s,17r)-3,7,12-trihydroxy-10,13-dimethyl-2,3,4,5,6,7,8,9,11,12,14,15,16,17-tetradecahydro-1h-cyclopenta[a]phenanthren-17-yl]pentanoate	*Niu Huang*	32.32	0.76
2	MOL008839	methyl deoxycholate	*Niu Huang*	34.63	0.73
3	MOL008845	deoxycholic acid	*Niu Huang*	40.72	0.68
4	MOL008846	zinc01280365	*Niu Huang*	46.38	0.49
5	MOL000953	CLR	*Niu Huang*	37.87	0.68
6	MOL001241	O-acetyl-α-boswellic acid	*Ru Xiang*	42.73	0.7
7	MOL001215	tirucallol	*Ru Xiang*	42.12	0.75
8	MOL001255	boswellic acid	*Ru Xiang*	39.55	0.75
9	MOL001243	3alpha-hydroxy-olean-12-en-24-oic-acid	*Ru Xiang*	39.32	0.75
10	MOL001295	phyllocladene	*Ru Xiang*	33.4	0.27
11	MOL001001	quercetin-3-o-β-d-glucuronide	*Mo Yao*	30.66	0.74
12	MOL001002	ellagic acid	*Mo Yao*	43.06	0.43
13	MOL001004	pelargonidin	*Mo Yao*	37.99	0.21
14	MOL001006	poriferasta-7,22e-dien-3beta-ol	*Mo Yao*	42.98	0.76
15	MOL001009	guggulsterol-vi	*Mo Yao*	54.72	0.43
16	MOL001013	mansumbinoic acid	*Mo Yao*	48.1	0.32
17	MOL001026	myrrhanol C	*Mo Yao*	39.96	0.58
18	MOL001028	(8r)-3-oxo-8-hydroxy-polypoda - 13e,17e,21-triene	*Mo Yao*	44.83	0.59
19	MOL001029	myrrhanones B	*Mo Yao*	34.39	0.67
20	MOL001031	epimansumbinol	*Mo Yao*	61.81	0.4
21	MOL001033	diayangambin	*Mo Yao*	63.84	0.81
22	MOL001040	(2r)-5,7-dihydroxy-2-(4-hydroxyphenyl)chroman-4-one	*Mo Yao*	42.36	0.21
23	MOL001045	(13e,17e,21e)-8-hydroxypolypodo-13,17,21-trien-3-one	*Mo Yao*	44.34	0.58
24	MOL001046	(13e,17e,21e)-polypodo-13,17,21-triene-3,18-diol	*Mo Yao*	39.96	0.58
25	MOL001049	16-hydroperoxymansumbin-13(17)-en-3β-ol	*Mo Yao*	41.05	0.49
26	MOL001052	mansumbin-13(17)-en-3,16-dione	*Mo Yao*	41.78	0.45
27	MOL001061	(16S, 20r)-dihydroxydammar-24-en-3-one	*Mo Yao*	37.34	0.78
28	MOL001062	15α-hydroxymansumbinone	*Mo Yao*	37.51	0.44
29	MOL001063	28-acetoxy-15α-hydroxymansumbinone	*Mo Yao*	41.85	0.67
30	MOL001095	isofouquierone [(5as,8ar,9r)-8-oxo-9-(3,4,5-trimethoxyphenyl)-5,5a,6,9-tetrahydroisobenzofurano[6,5-f][1,3]benzodioxol-8a-yl] acetate	*Mo Yao*	40.95	0.78
31	MOL001126		*Mo Yao*	44.08	0.9
32	MOL001131	phellamurin_qt	*Mo Yao*	56.6	0.39
33	MOL001138	(3r,20s)-3,20-dihydroxydammar- 24-ene	*Mo Yao*	37.49	0.75
34	MOL001156	3-methoxyfuranoguaia-9- en-8-one	*Mo Yao*	35.15	0.18
35	MOL001175	guggulsterone	*Mo Yao*	42.45	0.44
36	MOL000358	beta-sitosterol	*Mo Yao*	36.91	0.75
37	MOL000449	stigmasterol	*Mo Yao*	43.83	0.76
38	MOL000490	petunidin	*Mo Yao*	30.05	0.31
39	MOL000098	quercetin	*Mo Yao*	46.43	0.28
40	MOL000988	4,17(20)-(cis)-pregnadiene-3,16-dione	*Mo Yao*	51.42	0.48
41	MOL000996	guggulsterol IV	*Mo Yao*	33.59	0.74
42	5757	17-beta-estradiol	*She Xiang*	High	5
43	5880	3α-hydroxy-5β-androstan-17-one	*She Xiang*	High	5
44	8815	estragole	*She Xiang*	High	3
45	10409	normuscone	*She Xiang*	High	4
46	12452	androst-4,6-diene-3,17-dione	*She Xiang*	High	5
47	15818	3alpha,17-dihydroxy-5beta-androstane	*She Xiang*	High	5
48	77153	cyclotetradecan-1-one	*She Xiang*	High	4
49	184938	Hydroxymuscopyridine a	*She Xiang*	High	5
50	184939	hydroxymuscopyridine b	*She Xiang*	High	5
51	222865	5α-androstan-3,17-dione	*She Xiang*	High	5
52	441302	3beta-hydroxy-5alpha-androstan-17-one	*She Xiang*	High	5
53	446934	5 alpha-androstan-3,17-dione	*She Xiang*	High	5
54	5281670	morin	*She Xiang*	High	5
55	5316269	5-cis-cyclotetradecen-1-one	*She Xiang*	High	4
56	5316410	2,6-decamethylene pyridine	*She Xiang*	High	4
57	5319969	musclide a1	*She Xiang*	High	5
58	5319970	muscopyridine	*She Xiang*	High	4
59	5320193	2,6-nonamethylene pyridine	*She Xiang*	High	4
60	5351247	decamine	*She Xiang*	High	2
61	5701998	testosterone	*She Xiang*	High	5
62	7092676	5 beta-androstan-3 alpha, 17 beta-diol	*She Xiang*	High	5
63	9857253	5α-androstane-3β,17α-diol	*She Xiang*	High	5
64	9857254	5β-androstan-3α,17α-diol	*She Xiang*	High	5
65	9860744	3beta-hydroxy-androst-5-ene-17-one	*She Xiang*	High	5
66	11139246	muscol	*She Xiang*	High	4
67	11183661	5-cis-cyclopentadecen-1-one	*She Xiang*	High	4
68	11253089	muscone	*She Xiang*	High	4
69	12306765	3alpha-hydroxy-5alpha-androstan-17-one	*She Xiang*	High	5
70	12313579	N-nornuciferine	*She Xiang*	High	5
71	12358566	3β-hydroxy-androst-5-ene-17-one	*She Xiang*	High	5
72	16213098	alpha-estradiol	*She Xiang*	High	5
73	24867471	androsterone	*She Xiang*	High	5
74	54706645	3,5-dihydroxybenzoic acid	*She Xiang*	High	3
75	57336524	cyclovirobuxine	*She Xiang*	High	3
76	59853547	5 beta-androstan-3,17-dione	*She Xiang*	High	5

**FIGURE 3:**
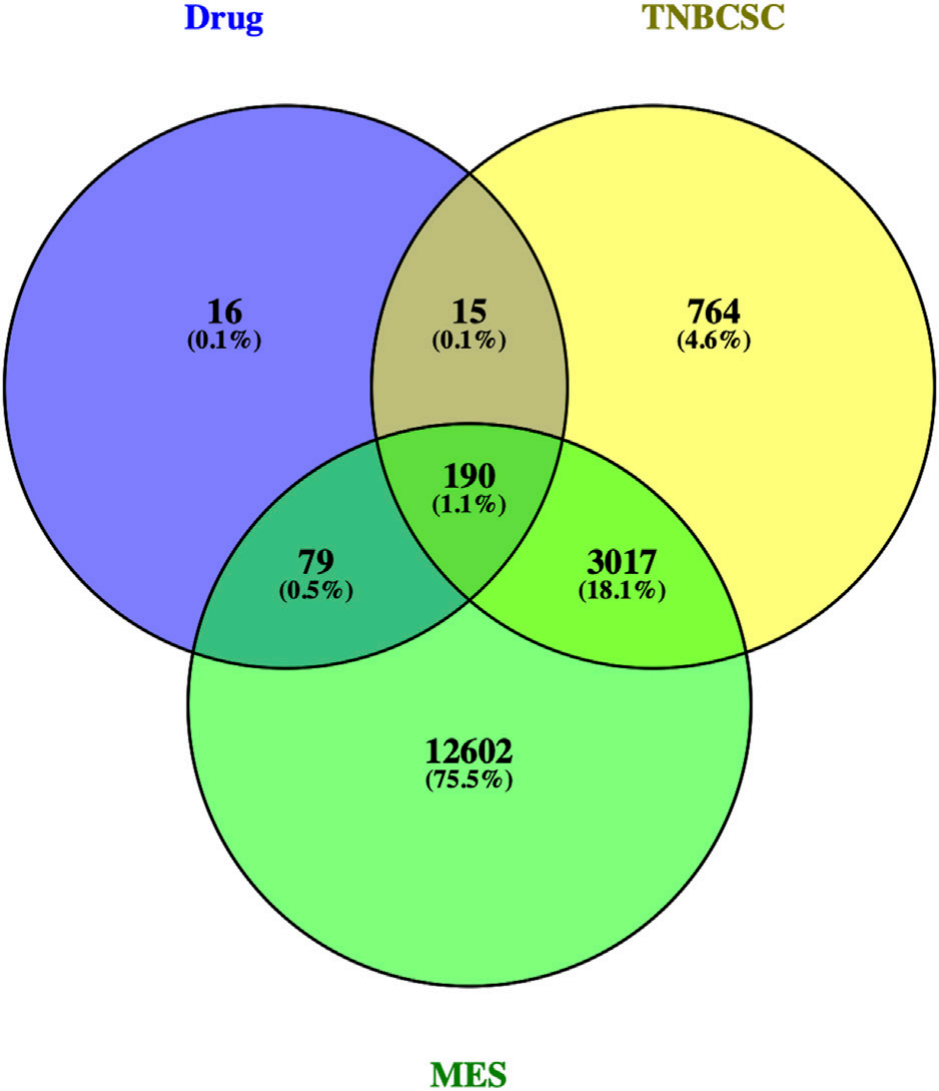
Venn diagram of the target genes for active compounds of Xihuang pill, TNBC stem cells, and a mesenchymal-like (MES) subtype of TNBC. Active compounds of Xihuang pill has 300 target genes, TNBC stem cell has 3,968 related target genes, and the mesenchymal-like (MES) subtype of TNBC has 15,888 target genes. There are 190 overlapping target genes among the three sets.

Three hundred protein targets of active compounds in Xihuang pill were predicted by TCMSP and the SwissTargetPrediction database; then, the protein names were transferred to gene symbols using the UniProt database. In total, 3,968 target genes related to TNBC stem cells were collected from the GeneCards and OMIM databases; 15,888 target genes in the gene sets of the MES subtype were obtained. Then, 190 overlapping target genes were recognized as candidate targets (Figure 3).

### Network Construction and Analyses

**FIGURE 4:**
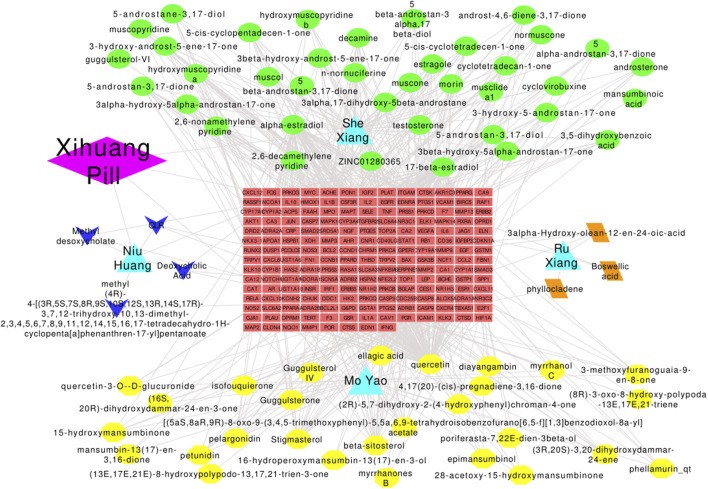
Drug–herbs–active compounds–target genes–disease network. Diamond: drugs; triangle: herbs; green-ellipse: active compounds of Shei Xiang; yellow-ellipse: active compounds of Mo Yao; arrow: active compounds of Niu Huang; parallelogram: active compounds of Ru Xiang; rectangle: target genes.

The drug–herb–active compound–target gene–disease network containing 268 nodes (1 drug, 76 active compounds, 190 target genes, and 1 disease) and 680 edges was established (Figure 4). Active components with high degree values may play an important role in the pharmacological effect of Xihuang pill. The top three components were quercetin (degree = 105), 17-beta-estradiol (degree = 60), and alpha-estradiol (degree = 60). Enrichment analyses such as GO enrichment analyses were conducted to study the biological characteristics of the 190 candidate targets. In total, 739 items with *p* values ≤ 0.05 were obtained, including 569 items relevant to BPs, 56 items relevant to CCs, and 114 items relevant to MFs. The top 15 most importantly enriched terms in the BP, CC, and MF categories are displayed in Supplementary Figure S1A. The results showed that the mechanisms of Xihuang pill targeting of TNBC stem cells were related to enzyme binding (BP), extracellular space (CC), and response to hypoxia (MF). In the KEGG enrichment analysis, there were 121 pathways with *p* values ≤ 0.05 (family-wise error correction), and the top 20 enriched pathways are shown in Supplementary Figure S1B. The pathways in cancer enriched most candidate targets (54 counts). The results indicated that pathways in cancer, hepatitis B, and the TNF signaling pathway were the significantly enriched pathways of Xihuang pill on the TNBC stem cells.

### Systems Biology Results and Molecular Docking

The CSCs are reported to be highly relevant to tumor metastasis, recrudescence, and therapy resistance (Chang, 2016; Doherty et al., 2017; Kuşoğlu and Biray Avcı, 2019). However, there are no universal CSC markers for each type of cancer to eradicate and target CSCs (Kuşoğlu and Biray Avcı, 2019). Gene expression microarray data (GSE10885) identified a group of MBCs that contrasted with the common breast tumor significantly expressed genes, which were highly related to TNBC stem cell–like properties (Hennessy et al., 2009).

**FIGURE 5:**
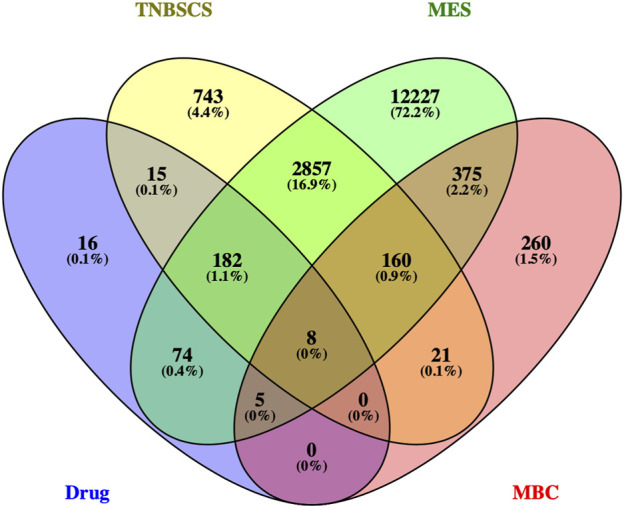
Venn diagram of the target genes for active compounds of Xihuang pill, TNBC stem cells, and mesenchymal-like (MES) subtype of TNBC and the significant genes of metaplastic breast cancer. Active compounds of Xihuang pill has 300 target genes, TNBC stem cell has 3,968 related target genes, the mesenchymal-like (MES) subtype of TNBC has 15,888 target genes, and metaplastic breast cancer has 829 significant genes. There are eight overlapping target genes between the four sets: CHUK, CLDN4, ITGAM, JUN, NR3C2, PPARD, RASA1, and RUNX2.

**FIGURE 6:**
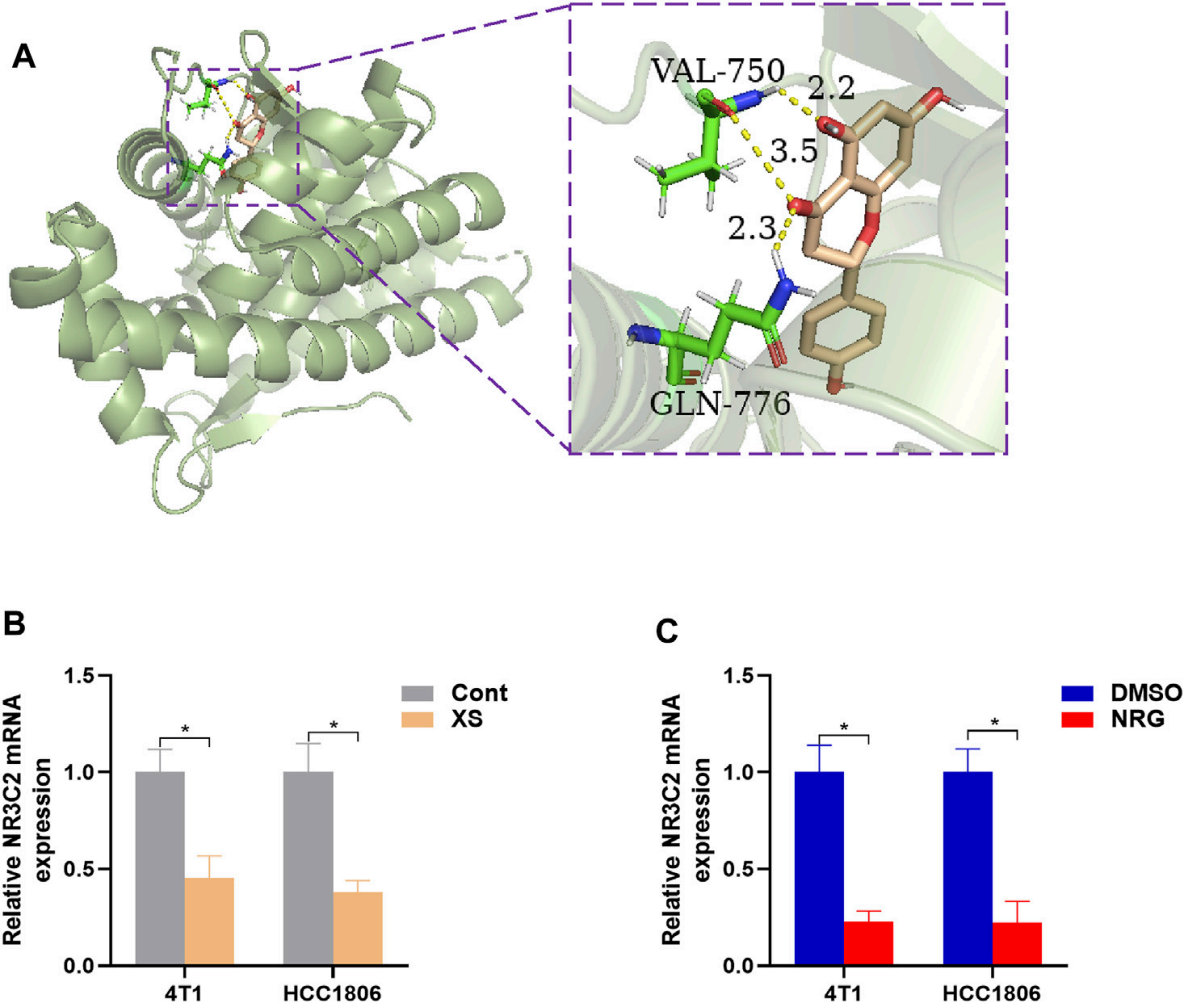
**(A)** The result of molecular docking of NR2C3 with naringenin. The smudge is target protein and mineralocorticoid receptor, and the target gene is NR3C2. Wheat: active compound, naringenin; green: binding sites; dotted yellow lines: bonds. The affinity value is −9.7 Kcal/mol. **(B,C)** RT-PCR outcomes, presented levels of NR3C2 after XS treatment compared to control serum (Cont) and 50 nM NRG treatment compared to control (DMSO). *p < 0.05, **p < 0.01 vs the control group.

Therefore, 829 significant genes were gathered from the analyses of gene expression microarray data (GSE10885). Then, the 829 significant genes intersected with 190 candidate targets, and eight key target genes were obtained: CHUK, CLDN4, ITGAM, JUN, NR3C2, PPARD, RASA1, and RUNX2 (Figure 5). The results of the molecular docking of eight key target proteins and their corresponding active compounds were obtained (Table 2). Based on the affinity, binding, binding sites, and text mining (Qin et al., 2011; Zhang et al., 2017; Zhao et al., 2018), the target gene NR3C2 and its active compound (2RR)-5,7-dihydroxy-2-(4-hydroxyphenyl)chroman-4-one (naringenin) were selected for further study (Figure 6A). It has been further confirmed that the level of NR3C2 has become lower after XS and NRG intervention vs the control group by RT-qPCR (Figures 6B,C).

**TABLE 2:** The result of molecular docking.

Target Genes	Compounds	Affinity (Kcal/mol)
CHUK	quercetin	−6.8
CLDN4	quercetin	−6.1
ITGAM	17-beta-estradiol	−9.7
ITGAM	alpha-estradiol	−9.4
JUN	beta-sitosterol	−5.2
JUN	quercetin	−5.8
NR3C2	methyl (4r)-4-[(3r,5s,7s,8r,9s,10s,12s,13r,14s,17r)-3,7,12-trihydroxy-10,13-dimethyl-2,3,4,5,6,7,8,9,11,12,14,15,16,17-tetradecahydro-1h-cyclopenta[a]phenanthren-17-yl]pentanoate	−8.4
NR3C2	deoxycholic acid	−8.3
NR3C2	CLR	−7.6
NR3C2	poriferasta-7,22e-dien-3beta-ol	−9.3
NR3C2	pelargonidin	−9.2
NR3C2	(2r)-5,7-dihydroxy-2-(4-hydroxyphenyl)chroman-4-one	−9.7
NR3C2	guggulsterone	−9.2
NR3C2	androst-4,6-diene-3,17-dione	−9.7
NR3C2	3beta-hydroxy-androst-5-ene-17-one	−8.4
NR3C2	cyclovirobuxine	−7.8
PPARD	quercetin	−8.5
RASA1	quercetin	No results
RUNX2	quercetin	No protein 3D structure

### NR3C2 Gene in the TCGA Data Set

**FIGURE 7:**
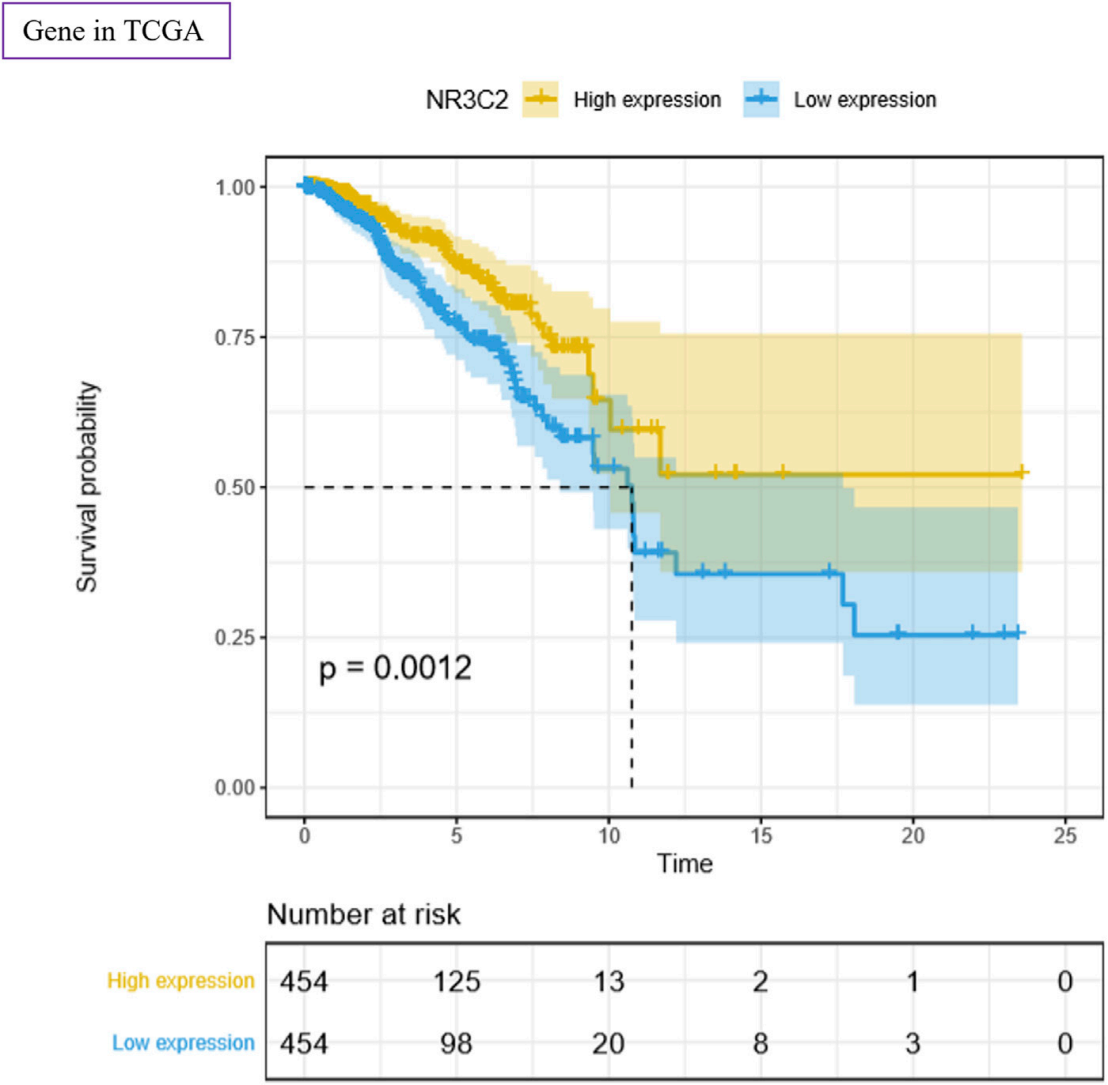
Survival analyses of NR3C2 regarding OS using the TCGA–BRCA data set. Yellow: high-expression group; blue: low-expression group. *p* value was log-ranked. Auto-selected best cutoffs were used.

To study the function of the NR3C2 gene in patients with TNBC, we used the TCGA–BRCA data set for analysis. The results showed a significant difference in survival probability regarding OS between the NR3C2 high-expression group and NR3C2 low-expression group (*p* = 0.0012) (Figure 7). The results suggested that the NR3C2 gene played an important role in TNBC, and a high expression of NR3C2 in patients with TNBC improved the OS. Additionally, compared with the NR3C2 low-expression group, patients with a high expression of NR3C2 showed obviously higher survival probability in stages I–IV (Supplementary Figures S2A–C), T1–4 (Supplementary Figures S2D–E), and M0 (Supplementary Figure S2G) (*p* < 0.05). The NR3C2 high-expression group in N0 had a higher survival probability, but not significantly (*p* = 0.098) (Supplementary Figure S2F). All results demonstrate that a higher expression of the NR3C2 gene in patients with TNBC enhanced the survival probability.

### Effect of Naringenin on TNBC Cell Lines

**FIGURE 8:**
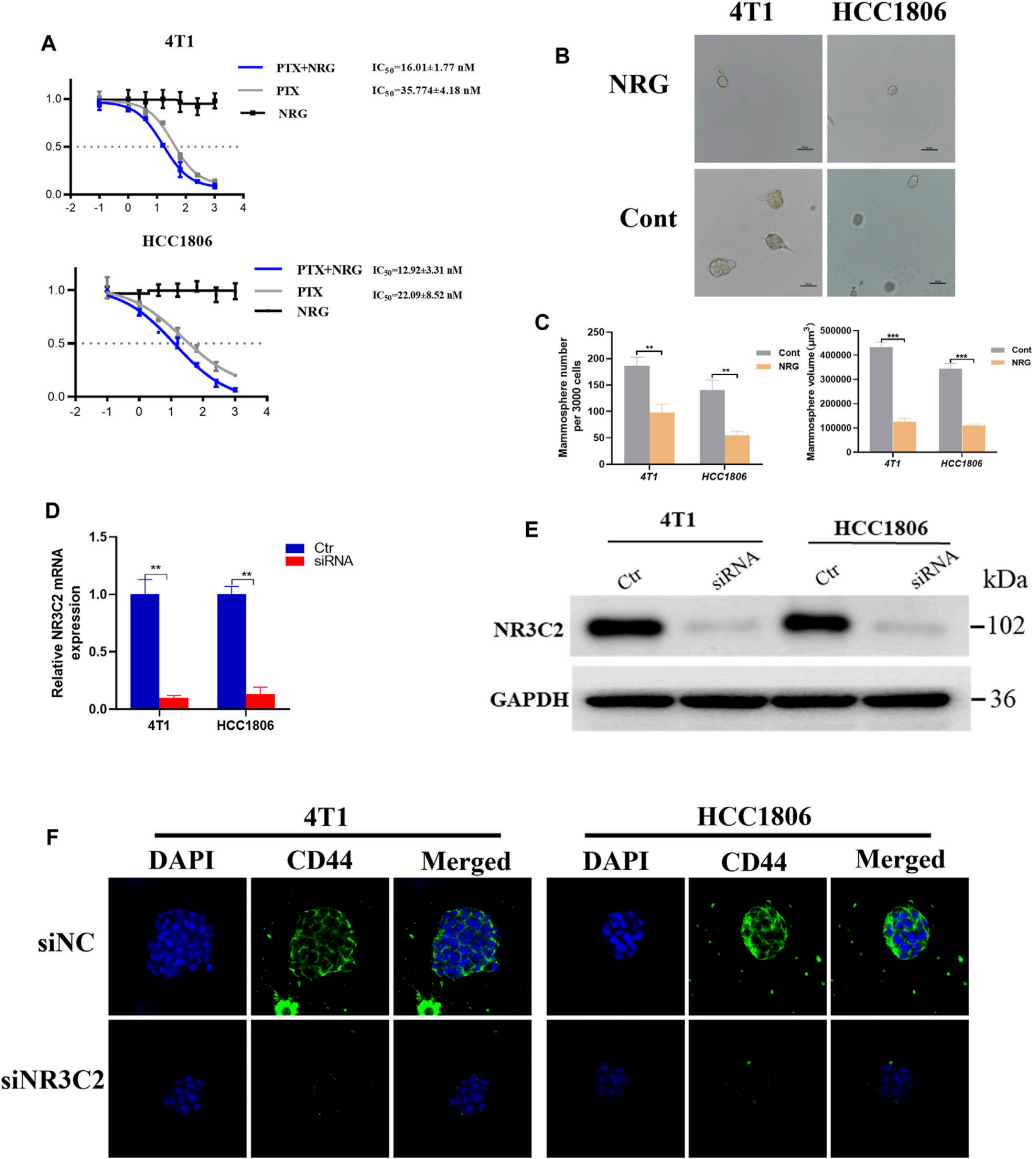
Effect of naringenin and NR3C2 on stem cell colony formation of TNBC cells. **(A)** 4T1 and HCC1806 cells were treated with 0, 0.1, 1, 4, 16, 64, 256, and 1024 nM PTX with or without 0, 0.1, 1, 4, 16, 64, 256, and1024 nM naringenin or 0, 0.1, 1, 4, 16, 64, 256, and1024 nM naringenin only for 48 h, respectively. The MTT assay was used to detect the viability of the cells (naringenin compared to control). **(B,C)** Representative photomicrographs of mammospheres formed by 4T1 and HCC1806 cells treated with naringenin and DMSO (Cont) are displayed. The quantification of the mammospheres number and size is presented as mean ± SD of three parallel experiments. Statistical analysis was conducted using two-way ANOVA. **(D,E)** RT-PCR and WB outcomes presented that the knockdown of NR3C2 caused decreasing protein expression levels of the mineralocorticoid receptor (MCR). *p < 0.05, **p < 0.01, ***p < 0.001 vs control. **(F)** Representative immunofluorescent images for siNC and siNR3C2 TNBC cells for CD44 (green) and DAPI (blue) signals.

An *in vivo* experiments of the relative percentage of naringenin and its metabolites in rats indicated that naringenin accounted for 72.2% of the production of metabolites in the blood plasma (Fan et al., 2017). To investigate the effect of naringenin on TNBC cells, we used the MTT assay and took photomicrographs. The MTT assays showed that naringenin itself could not directly cause fatal damage to 4T1 and HC1806 cells (Figure 8A). However, the outcomes showed that the IC_50_ value of PTX in the naringenin group was 16.01 ± 1.77 nM for 4T1 cells, while the IC_50_ value of the PTX-only group was 35.774 ± 4.18 nM (Figure 8A). For HCC1806 cells, the IC_50_ value of the PTX plus naringenin group was 12.92 ± 3.31 nM, while the IC_50_ value of the PTX-only group was 22.09 ± 8.52 nM (Figure 8B). These results demonstrated that for both 4T1 and HC1806 cells, IC_50_ values of PTX combined with naringenin had a lower IC_50_ value than that of the PTX-only groups, which indicated the effect of naringenin on improving the efficacy of PTX (Figures 8A,B). Then, photomicrographs were taken to explore the impact of naringenin on TNBC cells. Typical photomicrographs of mammospheres formed by 4T1 and HCC1806 cells are shown in Figure 8C. Compared with the control groups, the naringenin-treated 4T1 and HCC1806 cells formed fewer mammospheres, indicating that naringenin had an inhibitory effect on mammosphere formation in TNBC cells (4T1 and HCC1806 cells, Figure 8C).

### Effect of NR3C2 on TNBC Cell Lines

We analyzed the effect of the NR3C2 gene expression on patients with TNBC. Then, we probed the effect of the inhibited expression of the NR3C2 gene on 4T1 and HCC1806 cells. We examined the mRNA expression of NR3C2 in 4T1 and HCC1806 cells after the knockdown of the NR3C2 gene to validate that the transfections with NR3C2 vectors were successful. The outcomes demonstrated an obvious difference in the relative NR3C2 mRNA expression between knockdown groups and control groups, which indicated that the transfections of NR3C2 vectors succeeded (Figure 8D). Additionally, WB results showed decreased expression levels of the MCR protein encoded by NR3C2 genes in both 4T1 and HCC1806 cells (Figure 8E). TNBC cells' downregulation of NR3C2 by siRNA resulted in significant decrease of the breast cancer stem cell marker CD44 levels (Figure 8F). Then, the outcomes of MTT analyses showed that the IC_50_ values of TNBC cells treated with PTX were 31.56 ± 3.33 nM (4T1 cells) and 24.15 ± 2.45 nM (HCC1806 cells), while the IC_50_ values of siRNA groups treated with PTX were 29.36 ± 2.43 nM (4T1 cells) and 26.49 ± 2.78 nM (HCC1806 cells) (Table 3). The results indicate that the knockdown of NR3C2 did not change the effect of PTX on 4T1 and HCC1806 cells. The IC_50_ values of TNBC cells treated with PTX and naringenin were 13.25 ± 2.33 nM (4T1 cells) and 10.38 ± 3.46 nM (HCC1806 cells) (Table 3), respectively. Compared with 4T1 and HCC1806 cells treated with only PTX, naringenin improved the effect of PTX on these two TNBC cell lines. However, after knocking down the NR3C2 gene, the IC_50_ values of TNBC cells treated with PTX and naringenin were 32.13 ± 1.70 nM (4T1 cells) and 23.78 ± 2.47 nM (HCC1806 cells) (Table 3), which were not different from those of the control groups. The results show that the increasing effect of naringenin on the efficacy of PTX on 4T1 and HCC1806 cells was highly related to the expression level of the NR3C2 gene. Therefore, naringenin may target the NR3C2 gene to enhance the efficacy of PTX in 4T1 and HCC1806 cells.

**TABLE 3:** Half maximal inhibitory concentration (IC50) of groups cells treated with PTX.

	PTX(nM)	
	4T1	HCC1806
Ctr	31.56 ± 3.33	24.15 ± 2.45
siRNA	29.36 ± 2.43	26.49 ± 2.78
Ctr + NRG	13.25 ± 2.33	10.38 ± 3.46
siRNA + NRG	32.13 ± 1.70	23.78 ± 2.47

## Discussion

Breast cancer is one of the most lethal cancers in women worldwide. TNBC is a subtype of breast cancer with a higher risk of distant metastasis and recurrence and still lacks effective therapy. CSCs greatly contribute to the poor prognosis and unsatisfactory effect of chemotherapy. Due to the properties of TNBC, Chinese herbs and formulas are usually used as supplementary drugs, one of which is Xihuang pill. Compared with Western medicine, instead of targeting only one target, Chinese formulas treat diseases by regulating multiple targets and pathways. However, this characteristic of Chinese formulas makes it more difficult to study the mechanisms in depth. Network pharmacology is a new and useful tool to study the complex mechanisms of Chinese formulas. The current study used this tool to examine the underlying mechanisms of Xihuang pill in targeting TNBC stem cells.

One of the difficulties in pharmacological research of TCM is the unclear active ingredients. In this study, we orally administered Xihuang pill to rats and used the serum of those rats to study the effect of Xihuang pill on breast cancer cell lines, which was consistent with the metabolic process of TCM in the human body and was a more direct and effective research on the mechanism of Chinese formulas. Additionally, in the network pharmacological study, databases were used to perform ADME screening to select active compounds that were considered to be absorbed and circulated in the human body. To increase accuracy, we collected TNBC stem cell–related targets from databases and performed text mining. Jiang et al. classified TNBCs into four transcriptome-based subtypes: the basal-like and immune-suppressed (BLIS) subtype, the luminal androgen receptor (LAR) subtype, the immunomodulatory (IM) subtype, and the mesenchymal-like (MES) subtype (Jiang et al., 2019). The MES subtype was characterized by enrichment in the mammary stem cell pathways, which is highly related to our study. Hence, we used candidate gene targets that were recognized within the MES subtype to select the gene targets on the TNBC stem cells to enhance the precision. Then, we classified 190 overlapping genes of drug targets, disease targets from databases, and disease targets from text mining as candidate targets. In the drug–herb–active compound–target gene–disease network, active targets and targets with a higher degree may be more essential in treating TNBC. The top three active compounds are quercetin, 17-beta-estradiol, and alpha-estradiol. The GO enrichment analysis results indicate that enzyme binding (BP), extracellular space (CC), and response to hypoxia (MF) may be vital in the regulation of the Xihuang pill targeting of TNBC stem cells. Additionally, the outcomes of the KEGG enrichment analysis show that the candidate targets were mainly enriched pathways in cancer, hepatitis B, and the TNF signaling pathway. The drug–herb–active compound–target gene–disease network, PPI network, and enrichment analyses are useful tools to discover vital active compounds, drug targets, and pathways. However, network pharmacology still has limitations, such as the quality of databases and network algorithms and a lack of consistent and effective standards. Therefore, we added the systems biology result, gene expression microarray data (GSE10885) (Hennessy et al., 2009), and molecular docking to further filter the gene targets. Hennessy et al. reported that in contrast to other breast cancers, most metastasis breast cancers (MBCs), with stem cell–like features of aggressive, chemoresistant, and poor outcomes, displayed a significant similarity to a ‟tumorigenic” signature defined using CD44(+)/CD24(−) breast tumor-initiating stem cell-like cells (Hennessy et al., 2009). Hence, the systems biology result GSE10885 of significantly expressed genes of patients with MBCs vs patients with common breast tumor was used to select targets related to stem cell–like properties. The affinity of ITGAM and 17-beta-estradiol, NR3C2 and naringenin, and NR3C2 and Androst-4,6-diene-3,17-dione was the lowest which was −9.7. Besides, considering the bindings and binding sites, we found that NR3C2 and naringenin had the best docking. Thus, the target NR3C2 gene and its corresponding active compound naringenin were chosen.

Nuclear receptor subfamily 3 group C member 2 (NR3C2), which is one of the nuclear hormone receptors in the NR3C class, encodes the mineralocorticoid receptor (MR). For both glucocorticoids (GCs) and mineralocorticoids (MCs), the mineralocorticoid receptor regulates the function of aldosterone on the water and salt balance of restricted target cells (Arriza et al., 1987; Horisberger and Rossier, 1992). Several studies have indicated that NR3C2 can be a tumor suppressor gene in cancers such as colorectal, pancreatic, and cervical cancers (Fabio et al., 2007; Huang et al., 2011; Pesson et al., 2014; Yang et al., 2016). A lower expression of NR3C2 was suggested to be related to an increase in angiogenesis in early stage cervical carcinoma and poor prognosis in pancreatic cancer patients (Huang et al., 2011; Yang et al., 2016). However, there are few studies about the relationship between NR3C2 and breast cancer. In this study, we used the TCGA–BRCA data set to analyze the correlation between the expression of NR3C2 and the OS in patients with breast cancer. The results show that a high expression of NR3C2 in patients with breast cancer in all stages could improve the survival probability. We knocked down the NR3C2 gene expression in 4T1 and HCC1806 cells, and the WB results also showed a decreased level of MCR. MTT analyses of the NR3C2 gene–silenced 4T1 and HCC1806 cells treated with PTX with or without naringenin indicate that naringenin regulated the NR3C2 gene to enhance the efficacy of PTX in 4T1 and HCC1806 cells. According to the GO and KEGG analyses of the candidate targets, NR3C2 may participate in the treatment of TNBC through the steroid hormone–mediated signaling pathway and signal transduction in BP; receptor complex, endoplasmic reticulum membrane, nucleoplasm, and nucleus in CC; protein binding, steroid binding, zinc ion binding, transcription factor activity, and sequence-specific DNA binding in MF; and the aldosterone-regulated sodium reabsorption pathway. Further study of the specific and in-depth mechanism is required.

Naringenin, which is a flavonoid, is one of the active compounds in Mo Yao and has anti-inflammatory, antioxidant, and antitumor effects (Hernández-Aquino and Muriel, 2018; Zeng et al., 2018). Fan et al. (2017) studied the *in vivo* metabolic pathway of naringenin in rats with intragastric administration using LC-QTRAP-MS. The results showed that naringenin and its metabolites could be identified in plasma, bile, urine, and feces. Therefore, naringenin as an active compound in Myrrha could enter the systemic blood circulation and be metabolized. Several studies have indicated that the anticancer effects of naringenin include inhibiting the migration and invasion of some cancer cells by modulating the cell cycle, cellular apoptosis, and expression of EMT-related proteins (Han et al., 2018; Chen et al., 2019; Zhao et al., 2019). In the current study, naringenin was selected as a vital active compound. Then, we tested the effect of naringenin on the inhibition of TNBC cell viability. The results showed that naringenin itself did not have a direct fatal effect on 4T1 and HCC1806 cells. However, compared with the PTX-only groups, the combination of PTX with naringenin improved the antitumor effect on two TNBC cell lines. Additionally, the results indicated that naringenin could restrain mammosphere formation in 4T1 and HCC1806 cells, which implies that naringenin may inhibit TNBC stem cells. However, in this study, the concentration of naringenin in XS was not determined, which needs a further study in the future.

Our study focused on the effect of Xihuang pill on TNBC cell lines and used network pharmacology to help select the active compounds and gene targets. This research suggests that although Xihuang pill cannot directly kill TNBC cells, it can inhibit the formation of mammospheres in TNBC cells and enhance the efficacy of PTX in TNBC cells. Additionally, we have found that the key target NR3C2 plays a significant role in the effect of naringenin in enhancing the efficacy of PTX on 4T1 and HCC1806 cells, and naringenin has a similar effect to Xihuang pill on TNBC cell lines. However, our study is only a preliminary exploration research. The evidence provided is not sufficient, especially the lack of *in vivo* experimental data. Most importantly, the results of network pharmacology provide a method to explore the research. More experiments and studies are required to clarify the explicit relationship between naringenin and the NR3C2 gene and their specific actions and mechanisms.

## Conclusion

This study has detected that neither Xihuang pill nor naringenin can cause a lethal effect on TNBC cells, but they can improve the anticancer effect of PTX on TNBC cells. Additionally, Xihuang pill and naringenin inhibit mammosphere formation in 4T1 and HCC1806 cells. NR3C2 is the key gene, and naringenin is a vital active compound in Xihuang pill, which may play a vital role in inhibiting the CSCs in TNBC cells. Additionally, naringenin enhances the efficacy of PTX on 4T1 and HCC1806 cells by regulating the expression of the NR3C2 gene. These outcomes preliminarily verify the efficacy of Xihuang pill in treating TNBC and show that the important active compound naringenin can reduce the stemness of TNBC cells by regulating the NR3C2 gene to produce a synergistic effect on PTX, which may provide a new train of thought for the in-depth study of the mechanism.

## Data Availability

The raw data supporting the conclusion of this article will be made available by the authors, without undue reservation, to any qualified researcher.

## Supplementary Material

SUPPLEMENTARY FIGURE S1 Enrichment analysis of the 190 overlapping target genes. **(A)** Bar graph of the 15 most significant of gene ontology. Green, biological process; blue, cellular component; and red, molecular function. **(B)** Bubble diagram of the KEGG enrichment analysis. Y-axis label represents pathway and X-axis label represents RichFactor (RichFactor = amount of the genes enriched in the pathway/amount of all annotated genes enriched in the pathway). Size and color of the bubble represent amount of the genes enriched in the pathway and its enrichment significance. Pathways in cancer have the most genes enriched in the pathway and Bladder cancer has the highest RichFactor.

SUPPLEMENTARY FIGURE S2 Subgroup survival analyses of the NR3C2 gene regarding OS using the TCGA–BRCA data set. Yellow: high-expression group; Blue: low-expression group. **(A–C)**: Stage; **(D,E)**: Tumor; **(F)**: Node; **(G)**:Metastasis. *p* value was log-ranked. Auto-selected best cutoffs were used.

## Abbreviations

ATCC, American Type Culture Collection; BP, biological process; CC, cellular component; CSCs, cancer stem cells; DL, drug-likeness: DMEM: Dulbecco’s modified Eagle’s medium; DMSO, dimethyl sulfoxide; FBS, fetal bovine serum; GEO, gene expression omnibus; GO, gene ontology; GSEA, Gene Set Enrichment Analysis; IC_50_, half maximal inhibitory concentration; KEGG, Kyoto Encyclopedia of Genes and Genomes; MES, mesenchymal-like; MF, molecular function; MTT, 3-(4,5-dimethylthiazol-2-yl)-2,5-diphenyltetrazolium bromide; NCBI, National Center for Biotechnology Information; OB, oral bioavailability; OMIM, Online Mendelian Inheritance in Man; OS, overall survival; PTX, paclitaxel; RT–qPCR, quantitative reverse transcription PCR; TNBC, triple-negative breast cancer; TCM, traditional Chinese medicine; TCMSP, traditional Chinese medicine systems pharmacology database; WB, Western blot; XS, serum containing Xihuang pill.
