# Supplementary figures and images for: Network Pharmacology–Based Identification of Key Mechanisms of Xihuang Pill in the Treatment of Triple-Negative Breast Cancer Stem Cells
Source: Front Pharmacol. 2021 Oct 19;12:714628. doi: 10.3389/fphar.2021.714628 (PMC8560791; doi:10.3389/fphar.2021.714628)

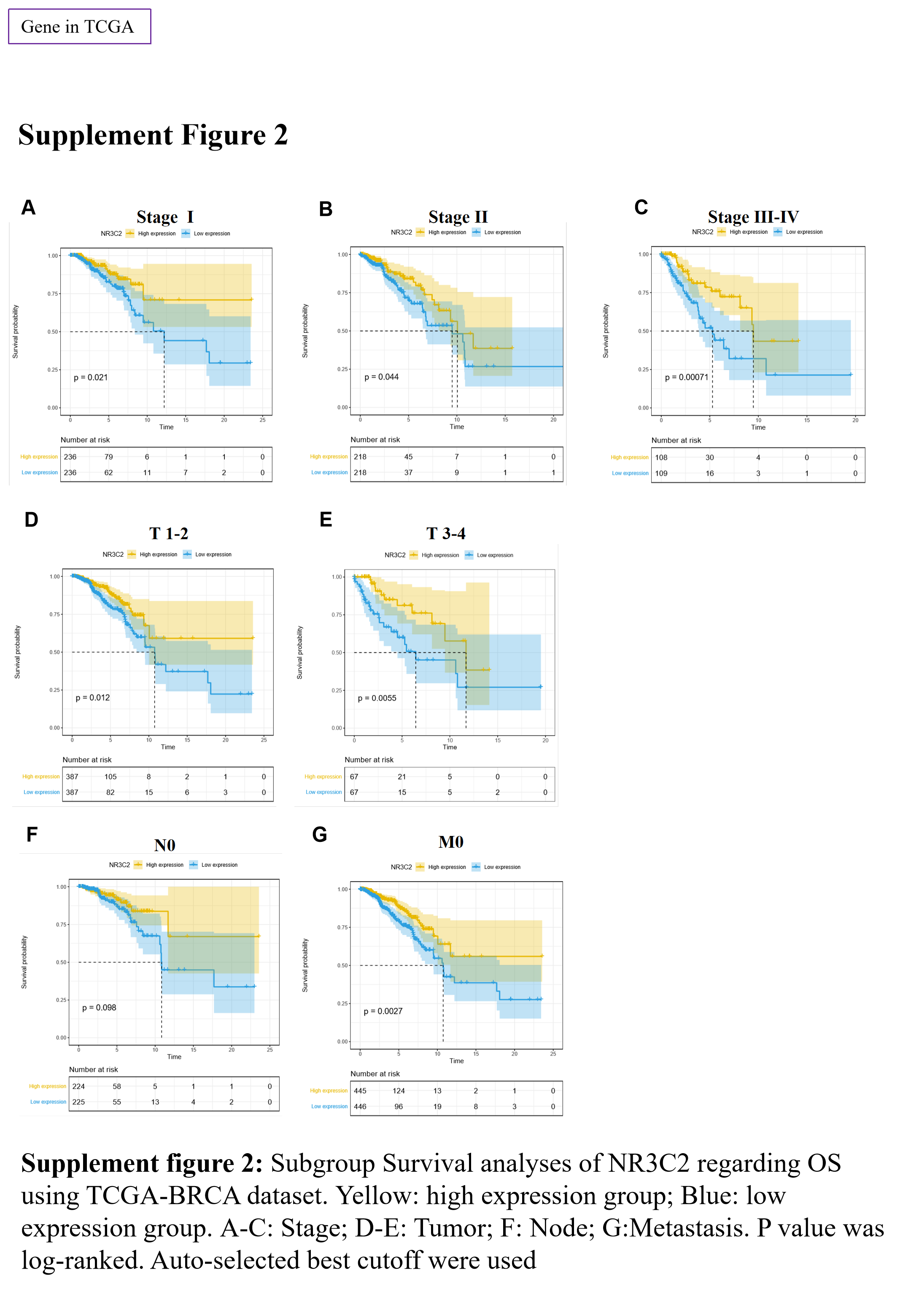

Supplement: Supplementary file 1 [file Image2.TIF]

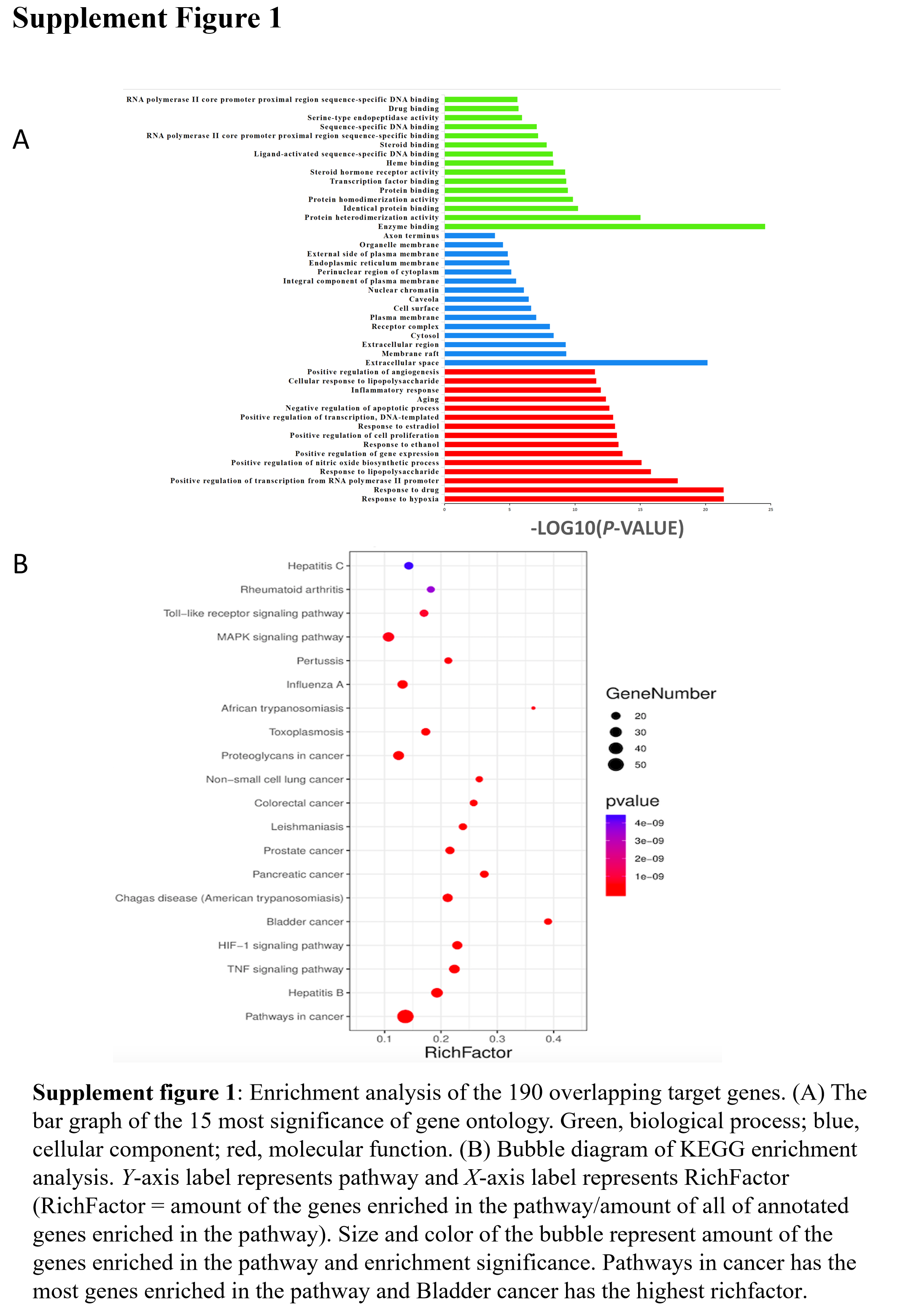

Supplement: Supplementary file 2 [file Image1.TIF]
